# ZrMOF nanoparticles as quenchers to conjugate DNA aptamers for target-induced bioimaging and photodynamic therapy†
†Electronic supplementary information (ESI) available. See DOI: 10.1039/c8sc02210k


**DOI:** 10.1039/c8sc02210k

**Published:** 2018-07-30

**Authors:** Yuan Liu, Weijia Hou, Lian Xia, Cheng Cui, Shuo Wan, Ying Jiang, Yu Yang, Qiong Wu, Liping Qiu, Weihong Tan

**Affiliations:** a Molecular Science and Biomedicine Laboratory (MBL) , State Key Laboratory of Chemo/Bio-Sensing and Chemometrics , College of Biology , College of Chemistry and Chemical Engineering , Hunan University , Changsha , 410082 , China . Email: tan@chem.ufl.edu ; Email: qiuliping@hnu.edu.cn; b Institute of Molecular Medicine , Renji Hospital , School of Medicine and College of Chemistry and Chemical Engineering , Shanghai Jiao Tong University , Shanghai 200240 , PR China; c Department of Chemistry and Department of Physiology and Functional Genomics , Center for Research at the Bio/Nano Inter-face , Health Cancer Center , UF Genetics Institute , McKnight Brain Institute , University of Florida , Gainesville , FL 32611-7200 , USA

## Abstract

Porphyrinic metal–organic framework (MOF) nanoparticles for photodynamic therapy solve the photosensitizer problems of poor solubility, self-quenching and aggregation. However, their low selectivity towards malignant tissues is an obstacle for bioimaging and a bottle-neck to cellular uptake for highly efficient photodynamic therapy of cancer. Here, ZrMOF nanoparticles as quenchers to conjugate DNA aptamers were developed for target-induced bioimaging and photodynamic therapy. A phosphate-terminal aptamer prepared by solid-phase DNA synthesis was anchored on the surface of ZrMOF nanoparticles through strong coordination between phosphate and zirconium. Based on π–π stacking-induced quenching of TAMRA by ZrMOF nanoparticles, target-induced imaging is achieved due to the structural change of the aptamer upon binding with the target. Aptamer-conjugated ZrMOF nanoparticles with target binding ability significantly enhanced the photodynamic therapy effect. Furthermore, phosphate-terminal aptamer conjugation method can be generalized to other types of MOF nanomaterials, such as UiO-66 and HfMOF nanoparticles, which can be potentially used in biochemistry.

## Abstract 2

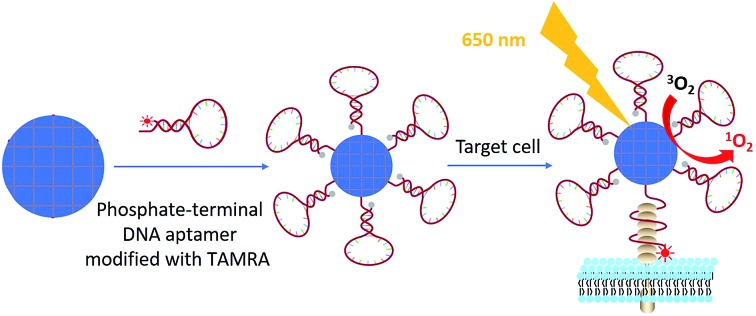
Aptamer conjugated porphyrinic metal–organic framework (MOF) achieved target-induced bioimaging and photodynamic therapy.

## Introduction

As emerging materials, nanoscale metal–organic frameworks (MOFs) are attracting intense interest in different areas such as energy storage,^1^–^3^ catalysis,^4^–^7^ and especially biochemical applications^8^–^11^ in sensing, nanomedicine and bioimaging, due to their well-defined structure with unique physical and chemical properties.^12^ Photodynamic therapy for cancer treatment involves generation of reactive oxygen species (ROS) by irradiation of photosensitizers at the tumor site.^13^,^14^ As a key to this method, porphyrin and its derivatives as photosensitizers have frequently been a limiting factor due to poor solubility, self-quenching and aggregation issues.^15^ These problems can be overcome using metal–organic framework (MOF) nanoparticles with precise spatial control and monomeric form, in particular porphyrin-based MOFs,^16^ which are promising candidates for imaging contrast, drug delivery and photodynamic therapy.

Despite the success of nanoscale MOFs in biomedical science,^8^,^17^,^18^ conventional bioimaging with these materials usually involves loading dyes into porous MOF nanoparticles or preparing intrinsic fluorescent MOF nanoparticles for further imaging study.^19^,^20^ However, leakage of dye from porous MOF nanoparticles is a potential problem. Meanwhile, non-specific accumulation of dye-loaded MOF and intrinsically fluorescent MOF nanoparticles can cause strong background signals and fake imaging information.^21^ Target-induced bioimaging can significantly decrease the background and fake imaging information. Current cancer therapy with nanoscale MOFs mainly relies on passive targeting (enhanced permeability and retention (EPR) effect) to improve the specific accumulation of drug at tumor sites.^22^,^23^ However, the EPR effect is complex and strongly depends on the size, surface properties and circulation time of nanoparticles, and large nanoparticles may have limited extracellular diffusion. In addition, some well-designed nanoparticles with a good EPR effect can penetrate throughout large tumor tissues following systemic administration, possibly causing side effects.^24^ By conjugating nanoparticles with targeting ligands, such as small molecules, peptides, antibodies or aptamers, the nanoparticles can bind with cell-surface receptors and enter cells by receptor-mediated endocytosis, thus enhancing cellular uptake into cancer cells rather than increasing accumulation in the tumor.^13^,^25^–^29^

**Scheme 1:**
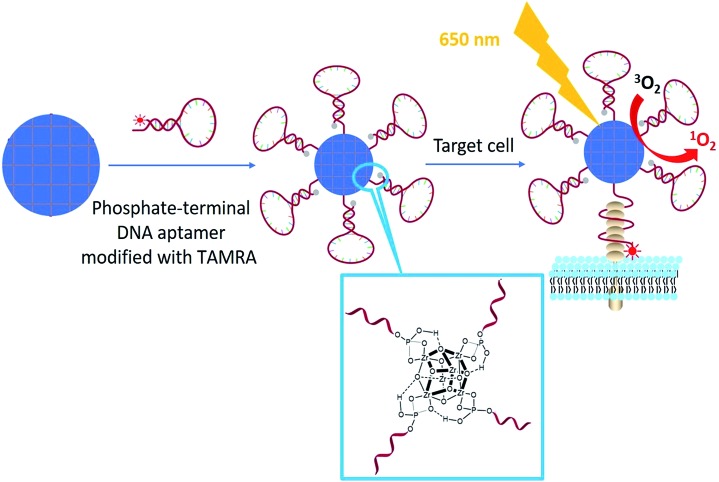
Illustration of phosphate-terminal DNA aptamer conjugation to a ZrMOF nanoparticle quencher for target-induced imaging and photodynamic therapy.

Although nanoscale MOF–DNA conjugates have been studied,^21^,^30^ their applications have been limited by complex organic synthesis before and post MOF construction, as well as as-synthesized organic linkers. We here developed general facile one-step aptamer conjugation to nanoscale MOFs for target-induced bioimaging and photodynamic therapy. Aptamers, selected from a large library by SELEX (Systematic Evolution of Ligands by Exponential Enrichment),^31^–^34^ are single-stranded oligonucleotides that can specifically bind to the target by folding into distinct secondary or tertiary structures. Phosphate, a functional group which has a strong coordination interaction with zirconium,^35^,^36^ was coupled to the 5′ end of an aptamer through solid-phase DNA synthesis for direct conjugation to zirconium-based MOF nanoparticles as shown in Scheme 1.

## Results and discussion

**Fig. 1:**
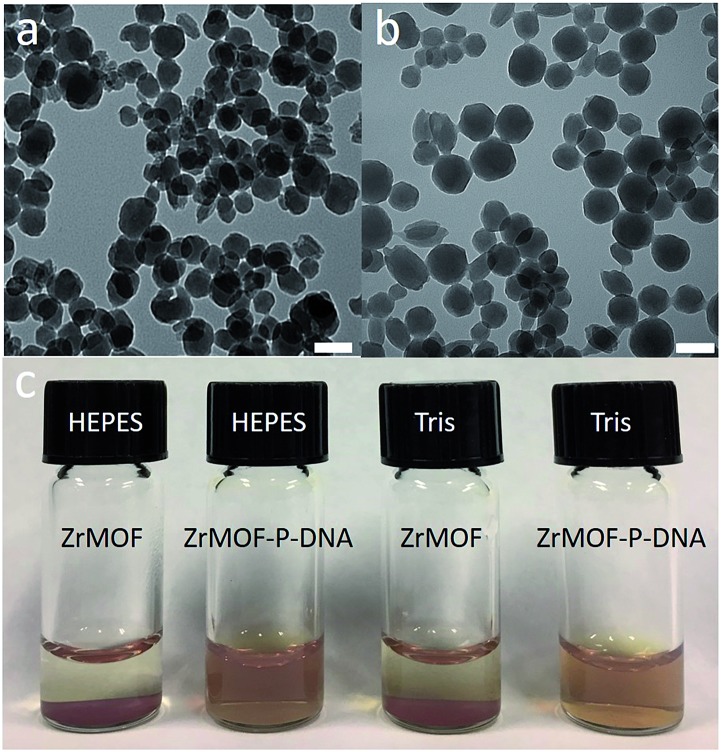
(a) Synthesized ZrMOF nanoparticles in DMF. (b) Aptamer conjugated ZrMOF nanoparticles (ZrMOF–P-aptamer) in water. (c) Stability test of ZrMOF nanoparticles and aptamer-conjugated ZrMOF nanoparticles in HEPES and Tris buffers. Scale bar: 100 nm.

Zr-based porphyrinic MOF (ZrMOF) nanoparticles were synthesized using ZrOCl_2_, tetra(4-carboxyphenyl)porphine (TCPP) and benzoic acid according to a literature report.^13^ X-ray diffraction confirmed the well-defined crystal structure of the as-synthesized ZrMOF nanoparticles (Fig. S1†). TEM indicated that the size of a ZrMOF nanoparticle is around 110 nm (Fig. 1a), and dynamic light scattering demonstrated a uniform size distribution of ZrMOF nanoparticles (Fig. S2†). To conjugate the aptamer, a phosphate-terminal aptamer was added to ZrMOF nanoparticles and incubated for 5 hours. The free aptamer was removed by washing with water and centrifugation. Before aptamer conjugation, ZrMOF nanoparticles showed a positively charged zeta-potential. After aptamer conjugation, a negatively charged zeta-potential was observed, because of the negatively charged DNA aptamer (Fig. S3†). DLS indicated a slight increase in size for ZrMOF nanoparticles after aptamer conjugation (Fig. S2†).

The stability of ZrMOF, as one of the most important features for biochemical study, was studied before and after DNA aptamer conjugation. Aptamer-conjugated ZrMOF nanoparticles showed much better stability after 24 hours than ZrMOF nanoparticles without any surface modification (Fig. 1c). Thus, phosphate-terminal DNA aptamer conjugation can significantly enhance the biostability of ZrMOF nanoparticles in buffers and increase their potential for biomedical applications.

**Fig. 2:**
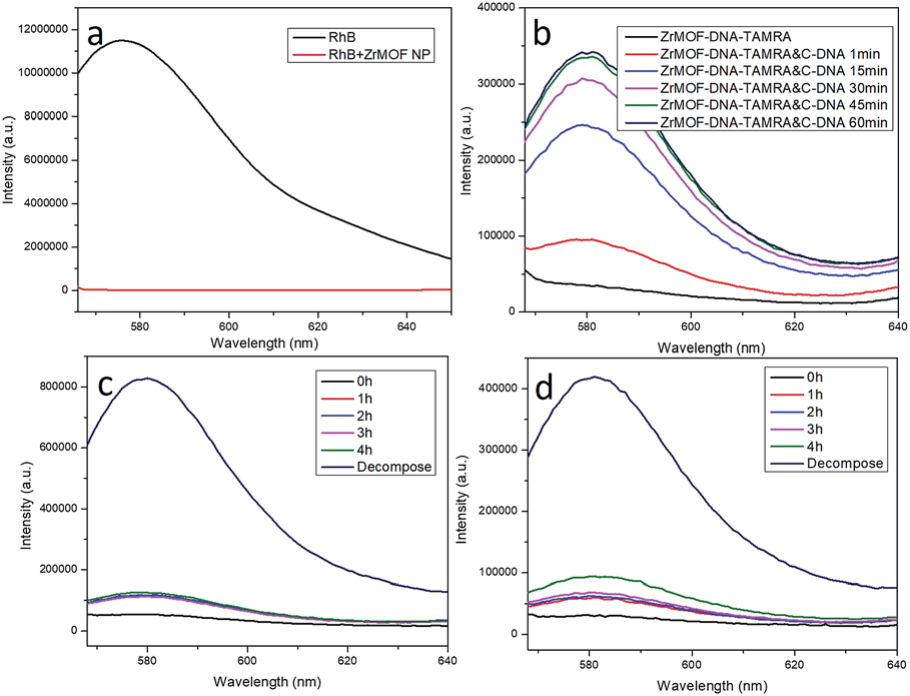
(a) Fluorescence of RhB was quenched upon addition of ZrMOF nanoparticles. (b) Fluorescence of TAMRA was quenched upon conjugation with ZrMOF nanoparticles but recovered after adding complementary DNA. (c) Quenching stability of ZrMOF–Library–TAMRA in cell culture medium DMEM. (d) Quenching stability of ZrMOF–aptamer–TAMRA in cell culture medium DMEM.

Having demonstrated that aptamer-conjugated ZrMOF nanoparticles are stable in different buffers, we next studied their utility in biomedical applications. In previous studies, nanoscale MOF nanoparticles were used as carriers for bioimaging by loading dyes into their well-defined porous structures. We here found that our ZrMOF nanoparticles can be used as quenchers for fluorescent dyes, such as RhB and TAMRA. As shown in Fig. 2a, the fluorescence of RhB was quenched upon adding to ZrMOF nanoparticles. This can be attributed to the conjugated π–π stacking effect between the TCPP linker and RhB *via* fluorescence resonance energy transfer (FRET). Similarly, the fluorescence of TAMRA was quenched as well when the TAMRA-modified aptamer was conjugated on the surface of ZrMOF nanoparticles, as shown in Fig. 2b. The fluorescence of TAMRA was recovered when the target complementary DNA (c-DNA) was added to hybridize with the TAMRA-modified aptamer to form a double-stranded DNA (Fig. 2b), thus detaching the TAMRA from the surface of ZrMOF nanoparticles, resulting in the recovery of fluorescence. This feature enabled us to construct a target-induced bioimaging system.

**Fig. 3:**
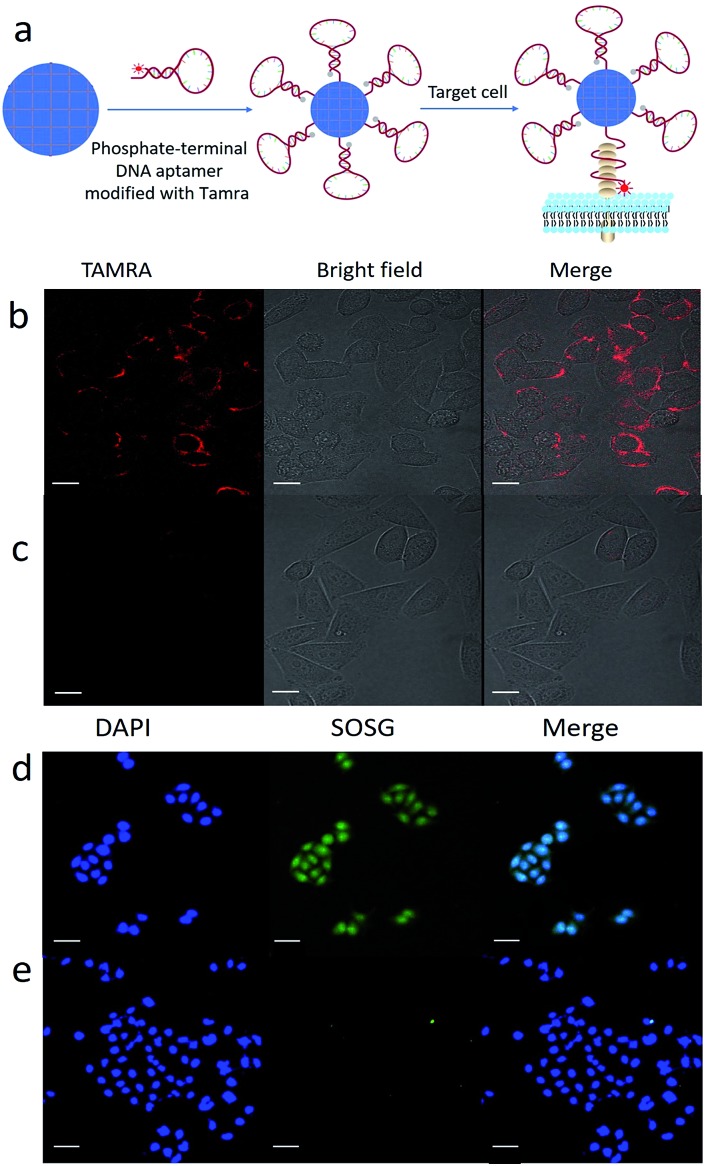
(a) Illustration of target-induced imaging with ZrMOF–aptamer–TAMRA. Aptamer structural change upon binding with the cell membrane receptor leads to fluorescence recovery. (b) Positive confocal imaging of HeLa cells incubated with ZrMOF–aptamer–TAMRA. (c) Negative confocal imaging of HeLa cells incubated with ZrMOF–Library–TAMRA. (d) Singlet oxygen detection right after treatment. A significant increase of ^1^O_2_ was observed after the cellular uptake of ZrMOF–aptamer and irradiation with a 650 nm laser. (e) Very few ^1^O_2_ were observed from the negative control group. Scale bar: (b) and (c) 20 μm; (d) and (e) 50 μm.

To validate the feasibility of target-induced bioimaging, we used ZrMOF nanoparticles conjugated with the TAMRA-modified DNA aptamer. As illustrated in Fig. 3a, the fluorescence of TAMRA was quenched after conjugating the aptamer to the surface of ZrMOF nanoparticles. But the fluorescence of TAMRA was recovered after binding with the target receptor on the cell membrane. Here, we selected the Sgc8 aptamer (Table S1†), which binds the target membrane protein PTK7 expressed on HeLa cells. Before conducting target-induced imaging, the fluorescence-quenching stability in Dulbecco's modified Eagle's medium (DMEM) culture medium was studied. No obvious fluorescence recovery was observed from ZrMOF–aptamer–TAMRA and ZrMOF–Library–TAMRA after four hours in HeLa cell culture medium DMEM (Fig. 2c and d), indicating a stable FRET under physiological conditions. The fluorescence of TAMRA was recovered when treated with 10× PBS buffer overnight, because the high concentration of free phosphate ions decomposed the ZrMOF nanoparticles and released the TAMRA modified DNA from the surface. A 2 hour incubation time of ZrMOF–aptamer–TAMRA and HeLa cells was used to study the target-induced imaging with ZrMOF–Library–TAMRA as a negative control. As shown in Fig. 3b, ZrMOF–aptamer–TAMRA exhibited an excellent target-induced imaging ability when incubated with HeLa cells, while ZrMOF–Library–TAMRA, which has a random DNA sequence and no target binding ability, showed negligible fluorescence in HeLa cells, as shown in Fig. 3c.

**Fig. 4:**
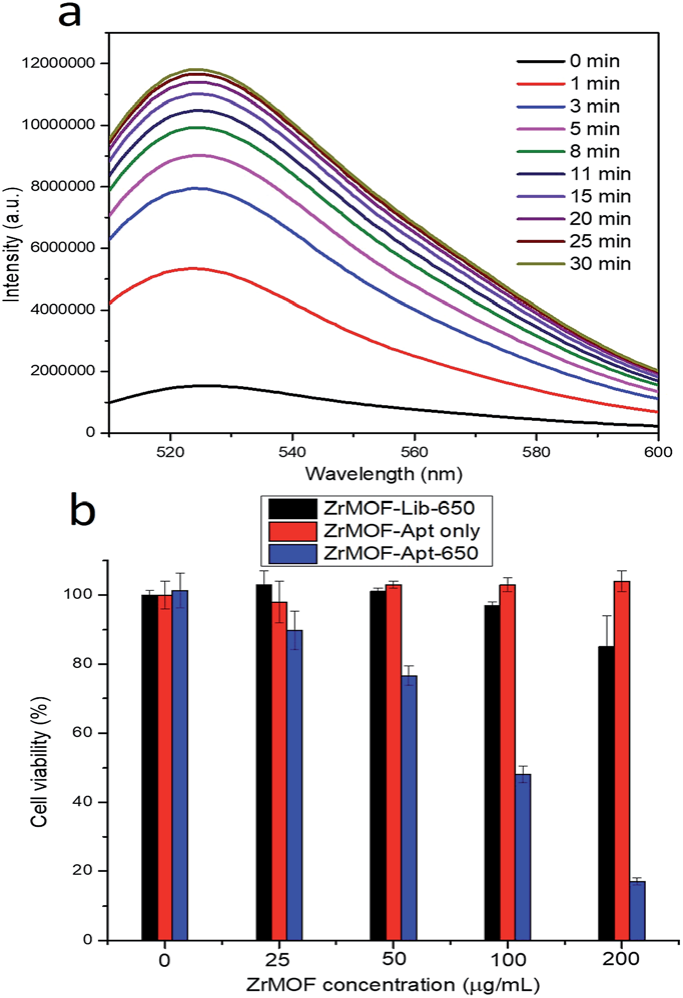
(a) Monitoring the singlet oxygen generation of ZrMOF–aptamer nanoparticles upon irradiating with a 650 nm laser (200 mW cm^–2^). (b) Cell viability study of ZrMOF–aptamer and ZrMOF–Library. For ZrMOF–Lib-650, HeLa cells were incubated with ZrMOF–Library nanoparticles and irradiated with a laser at 650 nm. For ZrMOF–Apt only, HeLa cells were incubated with ZrMOF–aptamer nanoparticles without laser irradiation. For ZrMOF–Apt-650, HeLa cells were incubated with ZrMOF–aptamer nanoparticles and irradiated with a laser at 650 nm. Laser power was 200 mW cm^–2^. Irradiation time was 5 min.

TCPP, the organic linker used in the synthesis of ZrMOF nanoparticles, and its derivatives have been used as photosensitizers for photodynamic therapy. However, their photodynamic therapy effect has been limited due to their hydrophobicity, aggregation tendency and insufficient selectivity to malignant tissues.^15^ To overcome these limitations, we took advantage of the aptamer-conjugated porphyrinic ZrMOF nanoparticles for targeted photodynamic cancer therapy. Porphyrinic ZrMOF nanoparticles absorb light at both the Soret band and Q band wavelengths (Fig. S4†). Irradiation at 650 nm, which can penetrate tissues, was selected to generate reactive oxygen species to kill the cancer cells. Singlet oxygen sensor green, which can be specifically oxidized by reactive oxygen species to produce enhanced fluorescence, was used as the ROS detector. Upon continuous laser excitation at 650 nm, porphyrinic ZrMOF–aptamer nanoparticles generated increasing amounts of singlet oxygen from 1 to 30 min (Fig. 4a). Moreover, the generation of singlet oxygen was confirmed with confocal imaging (Fig. 3d and e) of HeLa cells with ZrMOF–aptamer as the positive control and ZrMOF–Library as the negative control. A significant signal increase of singlet oxygen was observed when HeLa cells were treated with ZrMOF–aptamer (Fig. 3d). Photodynamic therapy of aptamer-conjugated porphyrinic ZrMOF nanoparticles was investigated by measuring cell viability using the MTS assay, as shown in Fig. 4b. HeLa cells were treated with ZrMOF–aptamer, ZrMOF–Library-650, and ZrMOF–aptamer-650 (irradiated with a 650 nm laser), respectively. ZrMOF–aptamer only without laser irradiation showed negligible cell death even at concentrations up to 200 μg mL^–1^ (red column). After laser irradiation (650 nm, 200 mW cm^–2^) for 5 min, ZrMOF–Library with a random DNA sequence and no target binding ability exhibited slight cell toxicity when the concentration was increased to 200 μg mL^–1^ (black column). However, significantly reduced cell viability was observed for HeLa cells when incubated with ZrMOF–aptamer nanoparticles under the same conditions as ZrMOF–Library nanoparticles (blue column). The cell viability was 85% for HeLa cells when treated with ZrMOF–Library at a concentration of 200 μg mL^–1^, while the cell viabilities were 48% and 17% for HeLa cells when treated with ZrMOF–aptamer at a concentration of 100 μg mL^–1^ and 200 μg mL^–1^, respectively. In addition, cell apoptosis analysis-based flow cytometry and live/dead cell staining also indicated that aptamer conjugation significantly increased the cell apoptosis efficiency (Fig. S8 and S9†). These results demonstrated that a higher photodynamic therapy effect can be achieved through targeting aptamer conjugation to ZrMOF nanoparticles.

**Fig. 5:**
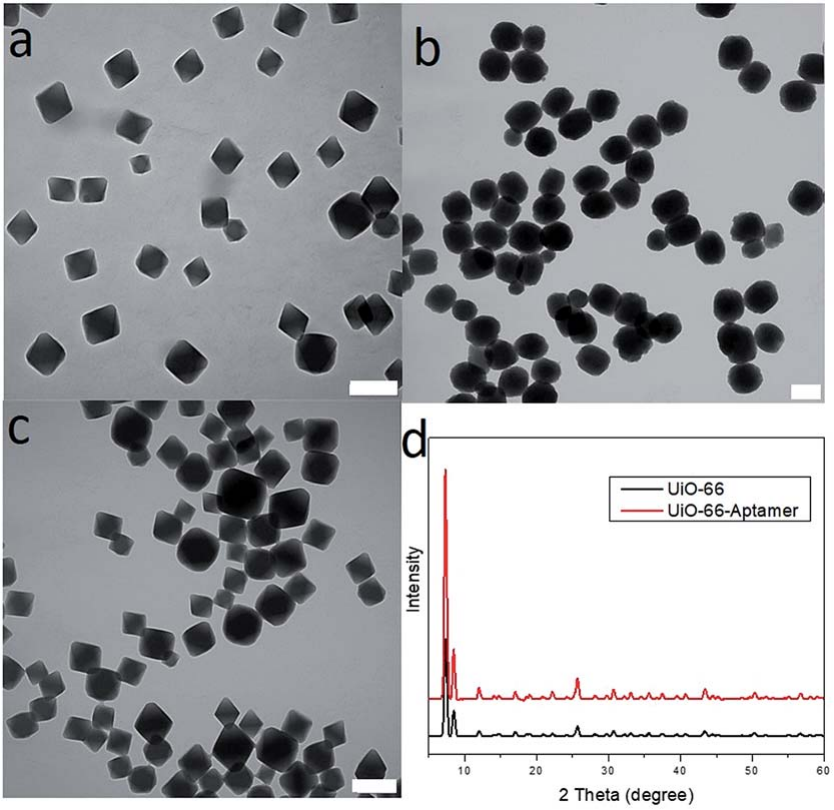
(a) TEM of UiO-66 nanoparticles before aptamer conjugation in DMF. (b) TEM of HfMOF nanoparticles after phosphate-terminal aptamer conjugation in water. (c) TEM of UiO-66 nanoparticles after phosphate-terminal aptamer conjugation in water. (d) Powder X-ray diffraction of UiO-66 nanoparticles before (black) and after phosphate-terminal aptamer conjugation (red). Scale bar: 200 nm.

The phosphate-terminal aptamer provided a facile strategy for DNA aptamer conjugation to ZrMOF nanoparticles. This method can be generalized to other types of MOF nanoparticles, such as UiO-66 and HfMOF nanoparticles (Fig. 5), which have potential applications in bioimaging and radiation therapy. As shown in Fig. S5,† UiO-66 nanoparticles were pink after conjugating with the phosphate-terminal aptamer modified with TAMRA. XRD results indicated that the crystal structure of UiO-66 did not change after conjugation with the phosphate-terminal aptamer modified with TAMRA (Fig. 5d). For HfMOF nanoparticles, their stability in water was significantly increased after conjugating with the aptamer through phosphate zirconium coordination (Fig. S6†).

## Conclusion

In conclusion, ZrMOF nanoparticles as quenchers to conjugate DNA aptamers for target-induced imaging and photodynamic therapy were developed and generalized to other types of MOF nanoparticles, such as UiO-66 and HfMOF. Target-induced imaging and targeted photodynamic therapy were achieved using ZrMOF nanoparticles as quenchers and photosensitizers, and an aptamer as a targeting ligand on the surface of ZrMOF nanoparticles. This facile aptamer conjugation to ZrMOF nanoparticles offers opportunities to develop MOF-based target-directed biosensors. On the basis of these superior features, we believe that future work can benefit from the rational design of engineering DNA aptamers and MOF nanomaterials for biomedical studies.

## Conflicts of interest

There are no conflicts to declare.

## Supplementary Material

Supplementary information
