# Supplementary material for: ZrMOF nanoparticles as quenchers to conjugate DNA aptamers for target-induced bioimaging and photodynamic therapy
Source: Chem Sci. 2018 Jul 30;9(38):7505–9. doi: 10.1039/c8sc02210k (PMC6179088; doi:10.1039/c8sc02210k)
Supplement: Supplementary file 1 [file SC-009-C8SC02210K-s001.pdf]

Electronic Supplementary Material (ESI) for Chemical Science.
This journal is © The Royal Society of Chemistry 2018

Supplemental Experimental Procedures

ZrMOF nanoparticles as quencher to conjugate DNA aptamer for target-induced bioimaging and photodynamic therapy

Yuan Liu, Weijia Hou, Lian Xia, Cheng Cui, Shuo Wan, Ying Jiang, Yu Yang, Qiong Wu, Liping Qiu and Weihong Tan

Materials: All reagents used were purchased from Sigma-Aldrich, unless otherwise stated. Meso-tetra(4-carboxyphenyl) porphine (TCPP) was purchased from Frontier Scientific. Benzoic acid, acetic acid and dimethylformamide (DMF) were purchased from Fisher. Singlet oxygen sensor green (SOSG) and fetal bovine serum were purchased from Invitrogen. DNA synthesis related reagents were purchased from Glen Research and Chem Gene. Dead Cell Apoptosis Kit with Annexin V Alexa Fluor® 488 & Propidium Iodide (PI) and Live/Dead cell staining kit were purchased from Thermo Fisher Scientific. The water used was purified on a Milli-Q Biocell System.

Synthesis of phosphate-terminal aptamer: the phosphate-terminal aptamer modified with TAMRA was synthesized using an ABI3400 DNA synthesizer (Applied Biosystems, Foster City, CA, USA), followed by addition of 2.5 mL of deprotection solution for 4 hours at 65 °C. NaCl solution (250 µL, 3 M) and cold ethanol (6 mL) were used to precipitate the deprotected phosphate-terminal aptamer. The precipitated aptamers were collected by centrifugation and redispersed in 400 µL of triethylammonium acetate for further purification by reversed-phase HPLC (Prostar, Varian, Walnut Creekk, CA, USA) using a C18 column. The purified aptamer was quantified by UV-Vis.

Synthesis of ZrMOF nanoparticles: In a typical synthesis, $ZrOCl_2$ (30 mg), TCPP (10 mg) and benzoic acid (280 mg) were dissolved in 10 mL of DMF in a 3-neck flask. The solution was incubated in an oil bath at 80°C for 5 hours. The resulting product was collected and washed with DMF and centrifugation for further aptamer conjugation.

Synthesis of UiO-66 nanoparticles: To synthesize UiO-66 nanoparticles, 1,4-benzenedicarboxylic acid (100 mg) was dissolved in 1 mL of DMF, and zirconyl chloride octahydrate (21 mg) was dissolved in 3 mL of DMF. The two solutions were then mixed together, followed by adding 2 mL of acetic acid. The resulting solution in the vial was incubated in an oil bath for 12 hours at 120 °C. UiO-66 nanoparticles were washed with DMF before further use.

Synthesis of HfMOF nanoparticles: To synthesize HfMOF nanoparticles, $HfCl_4$ (2 mg/mL in DMF) and TCPP (5 mg/mL in DMF) were prepared. Then 2 mL of $HfCl_4$, 2 mL of TCPP and 400 μL of acetic acid were mixed and added to a 3-neck flask. The resulting mixture was incubated in an 80 °C oil bath for 2 hours. After cooling, another 6 mL of DMF was added to the 3-neck flask and incubated another 24 hours. The resulting product was washed with DMF before further use.

Aptamer conjugation to ZrMOF (UiO-66, HfMOF) nanoparticles: A 300 μL aliquot of phosphate-terminal aptamer (100 μM) was added to freshly prepared ZrMOF nanoparticles in DMF (2mg/mL). The resulting solution was incubated for 5 hours at room temperature. Free aptamer was removed by washing with water and centrifugation. Aptamer conjugated ZrMOF nanoparticles were dispersed in water/buffer for further work.

Instruments and Characterization: Transmission Electron Microscopy (TEM) was performed using a Hitachi H-7000 transmission electron microscope at 100 kV. Twenty microliter prepared sample was dropped onto a carbon-coated copper grid (Ted Pella) and then dried for TEM.

The X-ray diffraction measurements was performed on a Bruker D8 ADVANCE diffractometer, employing the standard setup in reflection geometry.

Optical Absorption Spectroscopy. UV−vis absorption spectra were recorded using a Shimadzu UV-1800. Nanoparticles were dissolved in ethanol or water for measurement.

Dynamic light scattering (DLS) measurements were recorded on a Zetasizer Nano ZS, (Malvern Instrument Ltd., U.K.) equipped with a He−Ne laser operating at 633 nm at 25 °C.

Detecting singlet oxygen generation: To detect the singlet oxygen generation, singlet oxygen sensor green (SOSG) was introduced into a ZrMOF-aptamer nanoparticle solution using D_2O as solvent, followed by irradiation using 650 nm laser (200 mW/cm^2) for different periods of time. The fluorescence of SOSG was obtained with excitation at 494 nm after irradiation at different periods of time.

Intracellular singlet oxygen detection: HeLa cells were seeded in a 8-well cell cluture chamber and were incubated overnight. ZrMOF-Aptamer and ZrMOF-Library were incubated with HeLa cells for 3 hours fellowed by adding 10 μL of SOSG working solution and incubating for 30 min. Then, the cells were irradiated for 20 min (650 nm, 200 mW/cm^2). Subsequently, the incubation medium was removed and the cells were washed with PBS. The slide was mounted with DAPI mounting medium. Confocal images were collected with a Zeiss LSM 780 confocal microscope.

Internalization study: HeLa cells (60K) were planted in a confocal dish in 500 μL of Dulbecco's modified Eagle's medium (DMEM) supplemented with 10% FBS at 37 °C in 5% CO_2 for 24 hours to allow 70% confluence. Cells were then incubated with ZrMOF-Aptamer-TAMRA/ZrMOF-Library-TAMRA (25 μg/mL) in completed DMEM medium for 2 hours at 37 °C in 5% CO_2. The medium was then removed and the cells were washed twice with DMEM medium. Finally, confocal fluorescence images were acquired using a Leica TCS SP5 confocal microscope.

Cell viability study: A sample of 7000 HeLa cells in 100 μL of medium was seeded into each test well on a 96-well plate. After 24 hours culturing, supernatant was removed and ZrMOF-Library and ZrMOF-Aptamer nanoparticles in medium (150 μL) with different concentrations were added to each test well. The cells were incubated at 37 °C in a 5% CO_2 atmosphere for 6 hours. Then the supernatant was removed and 100 μL of fresh cell culture medium was added, followed by laser irradiation for 5 mins. After another 48 hours of incubation at 37 °C in a 5% CO_2 atmosphere, a standard MTS assay was performed. The absorbance value at 490 nm was determined by a microplate reader.

Live/Dead cell staining: Approximately 25,000 HeLa cells in 300 μL of medium was seeded into each test well on a 8-well cell culture chamber for an overnight incubation. Then ZrMOF-Lib, and ZrMOF-Apt were added and incubated for 6 hours at 37 °C in a 5% CO_2 atmosphere. Laser irradiation was applied for 5 mins after washing. After 24 hours'

incubation at 37 °C in a 5% CO_2 atmosphere, the Calcium AM/PI working solution was added and incubated for 30 min before collecting the fluorescence images.

Cell apoptosis by Alexa Fluor® 488 Annexin V/Dead Cell Apoptosis Kit: Approximatelly 100,000 HeLa cells in medium were seeded in a 24-well plate for an overnight incubation at 37 °C in a 5% CO_2 atmosphere. Then ZrMOF-Lib, and ZrMOF-Apt were added and incubated for 6 hours before appling a 5-min laser irradiation. The Alexa Fluor® 488 Annexin V/PI working solution was carried out by following the protocols. Finally, the flow cytometer was used to analyze the cell apoptosis.

Table S1: Detailed sequence information of DNA aptamer and library.

*: N is random base.

Name	Sequence
Phosphate-T10-Sgc8	5'-H_2PO_3-TTT TTT TTT TAT CTA ACT GCT GCG CCG CCG GGA AAA TAC TGT ACG GTT AGA-TAMRA-3'
Phosphate-T10-Sgc8-TAMRA	5'-H_2PO_3-TTT TTT TTT TAT CTA ACT GCT GCG CCG CCG GGA AAA TAC TGT ACG GTT AGA-TAMRA-3'
Phosphate-Library-TAMRA	5'-H_2PO_3-NNN NNN NNN NNN NNN NNN NNN NNN NNN NNN NNN NNN NNN NNN NNN NNN NNN NNN*-TAMRA-3'

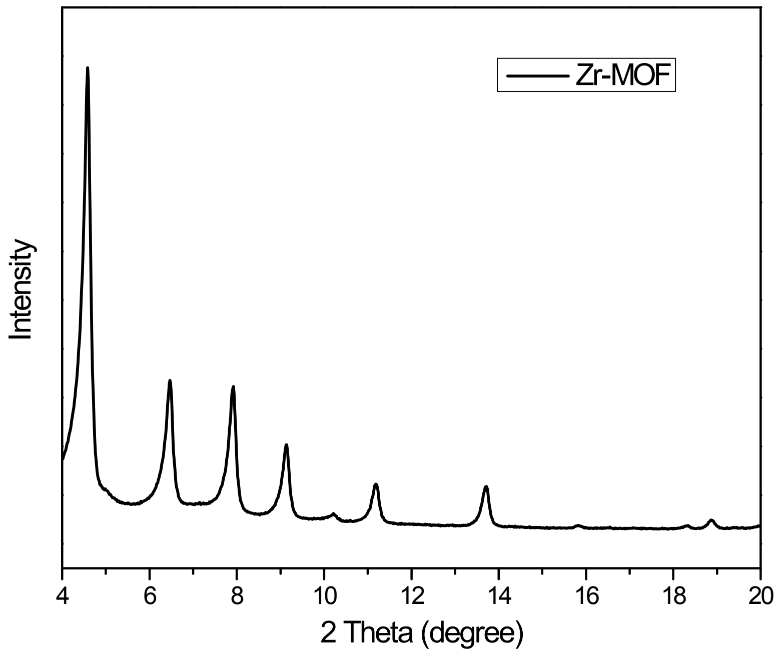

Figure S1. X-ray diffraction of as-synthesized ZrMOF nanoparticles.

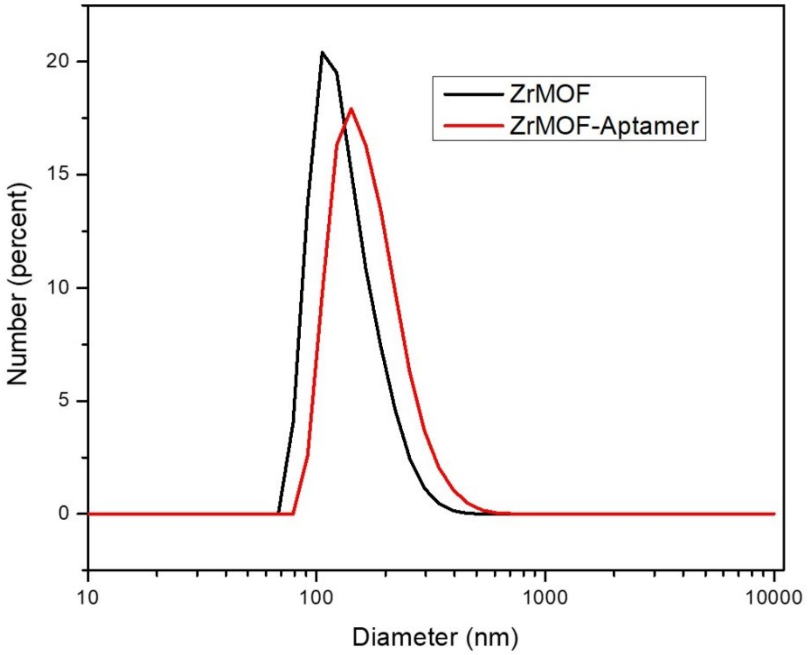

Figure S2. Dynamic light scattering of ZrMOF nanoparticles before and after phosphate-terminal aptamer conjugation.

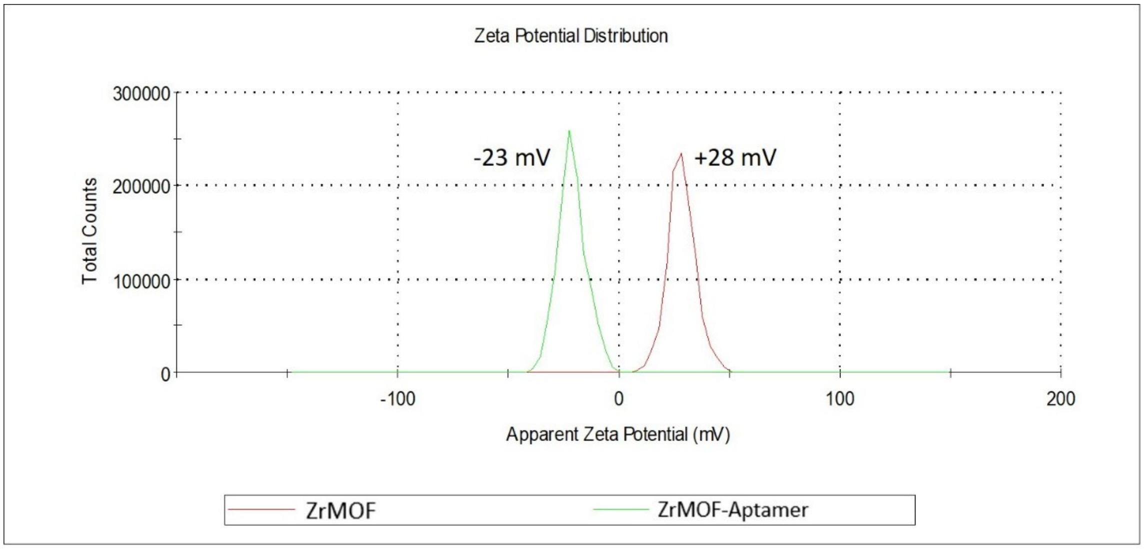

Figure S3. Zeta-potential of ZrMOF nanoparticles before (red) and after (green) conjugation with phosphate-terminal aptamer.

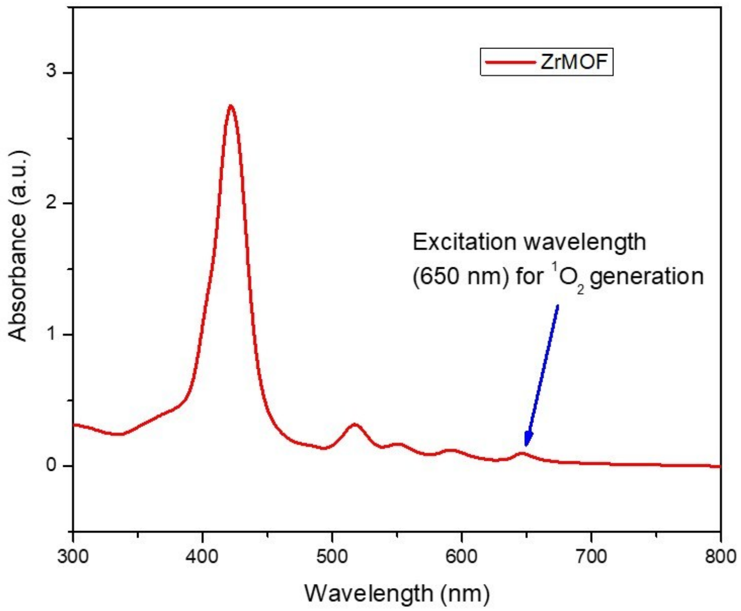

Figure S4. UV-Vis absorption of ZrMOF nanoparticles.

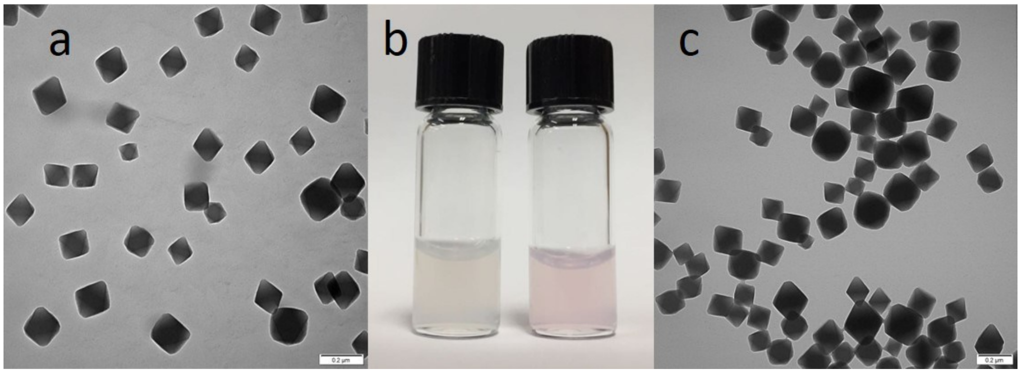

Figure S5. a) TEM of UiO-66 nanoparticles in DMF. b) Digital picture of UiO-66 nanoparticles before and after conjugation of phosphate-terminal aptamer modified with TAMRA (left: UiO-66 nanoparticles in DMF, right: UiO-66-aptamer in water). c) TEM of UiO-66 nanoparticles in water.

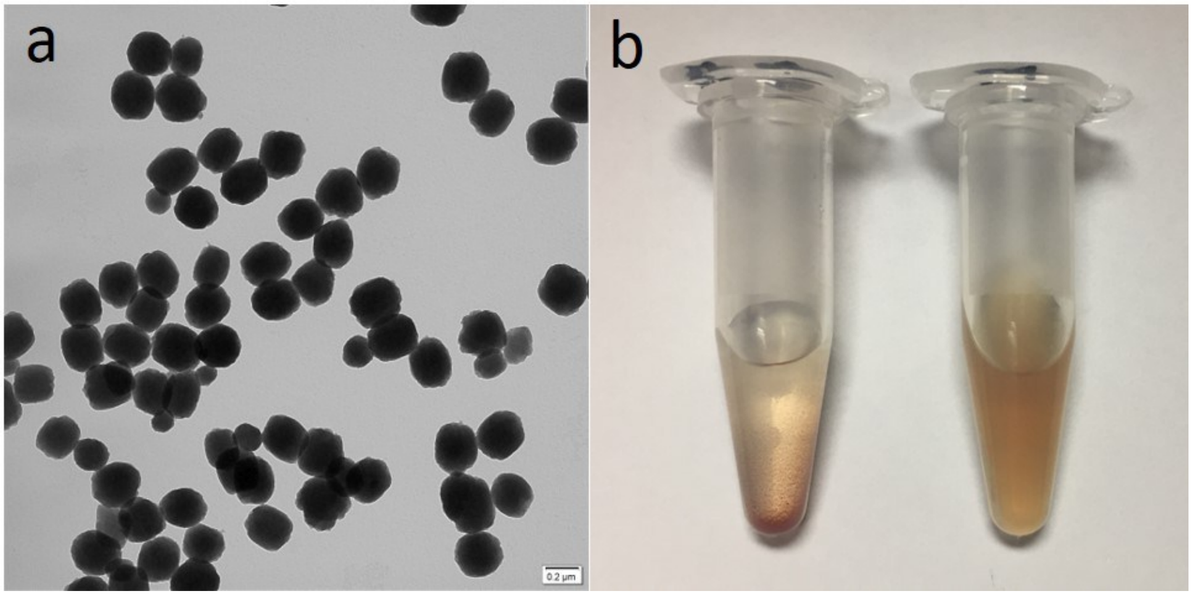

Figure S6. a) TEM of HfMOF nanoparticles. b) HfMOF nanoparticles before (left) and after phosphate-terminal aptamer conjugation (right) in water.

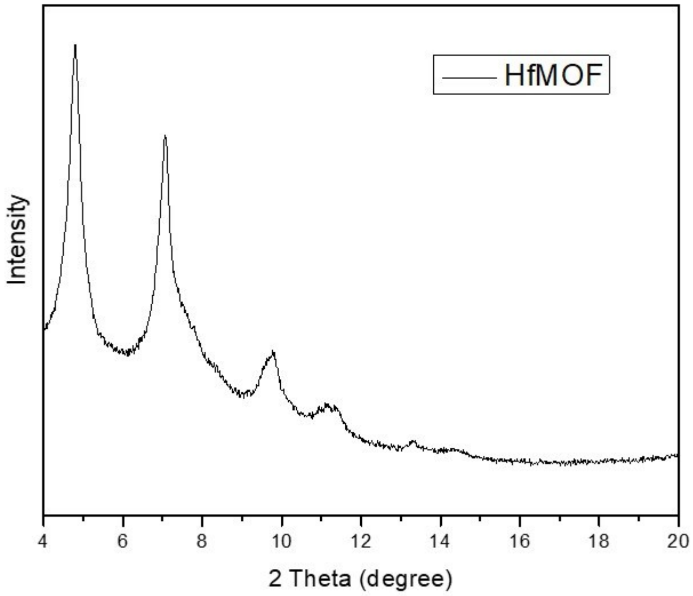

Figure S7. Powder X-ray diffraction of HfMOF nanoparticles.

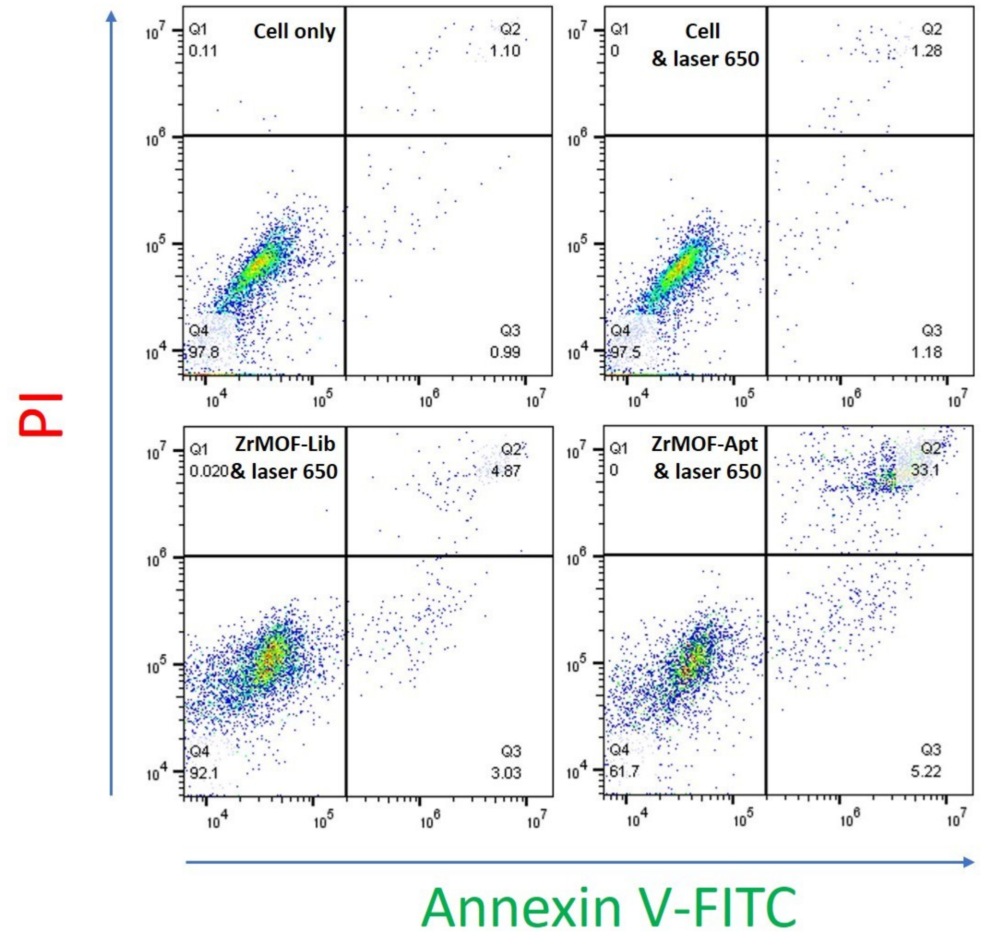

Figure S8. Cell apoptosis of HeLa cells after treatment with laser (650 nm), ZrMOF-Lib & laser (650), and ZrMOF-Apt & laser (650 nm).

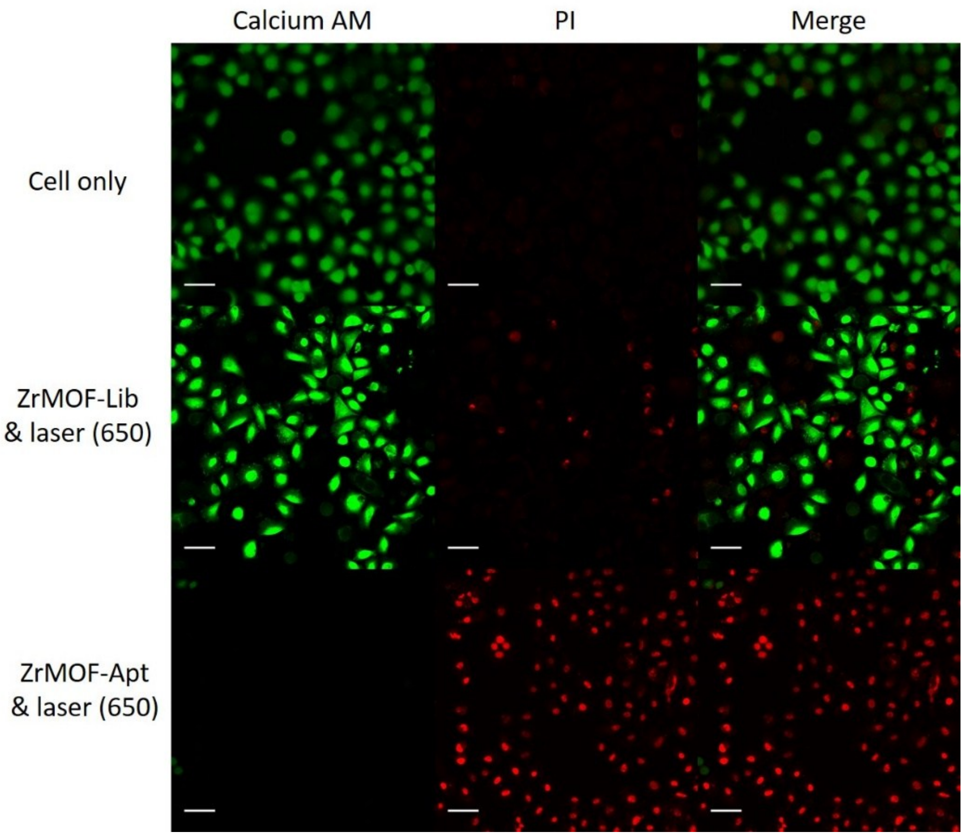

Figure S9. Live/Dead cell staining after treatment with ZrMOF-Lib & laser (650), and ZrMOF-Apt & laser (650 nm).
